# Correction: ErbB4 deletion in noradrenergic neurons in the locus coeruleus induces mania-like behavior via elevated catecholamines

**DOI:** 10.7554/eLife.44814

**Published:** 2019-01-08

**Authors:** Shu-Xia Cao, Ying Zhang, Xing-Yue Hu, Bin Hong, Peng Sun, Hai-Yang He, Hong-Yan Geng, Ai-Min Bao, Shu-Min Duan, Jian-Ming Yang, Tian-Ming Gao, Hong Lian, Xiao-Ming Li

Cao SX, Zhang Y, Hu XY, Hong B, Sun P, He HY, Geng HY, Bao AM, Duan SM, Yang JM, Gao TM, Lian H, Li XM. 2018. ErbB4 deletion in noradrenergic neurons in the locus coeruleus induces mania-like behavior via elevated catecholamines. *eLife* **7**:e39907. doi: 10.7554/eLife.39907.

Published 4, September 2018

In the published article, we inappropriately used the control and *TH-Cre;Erbb4^loxp/loxp^* groups of Figure 3E-G in Figure 2A-D to represent baseline difference between the two groups. This issue was noticed when rereading the published version. We have replaced Figure 2A-D with graphs generated from new data in the corrected version.

The corrected Figure 2 is shown below:

**Figure:**
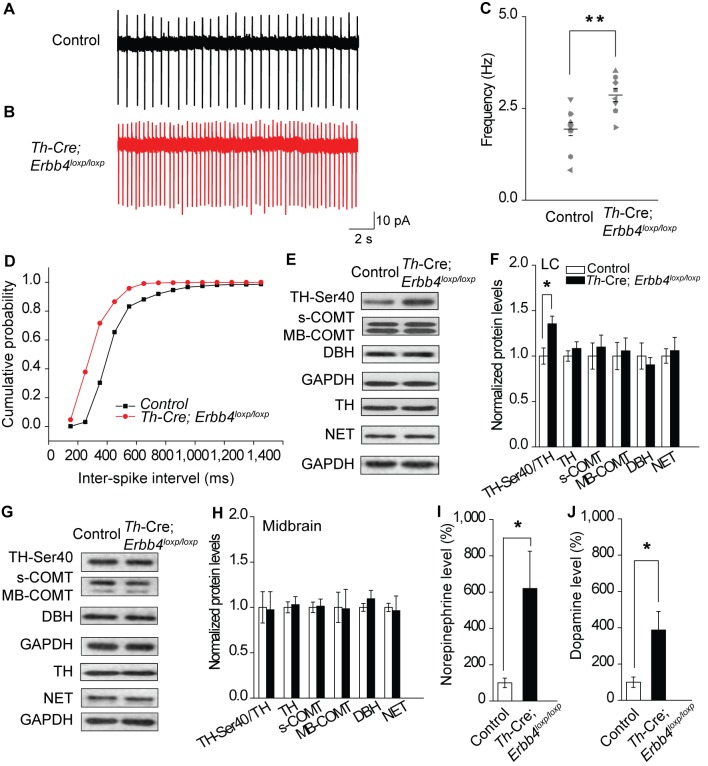


The original published Figure 2 for reference was:

**Figure:**
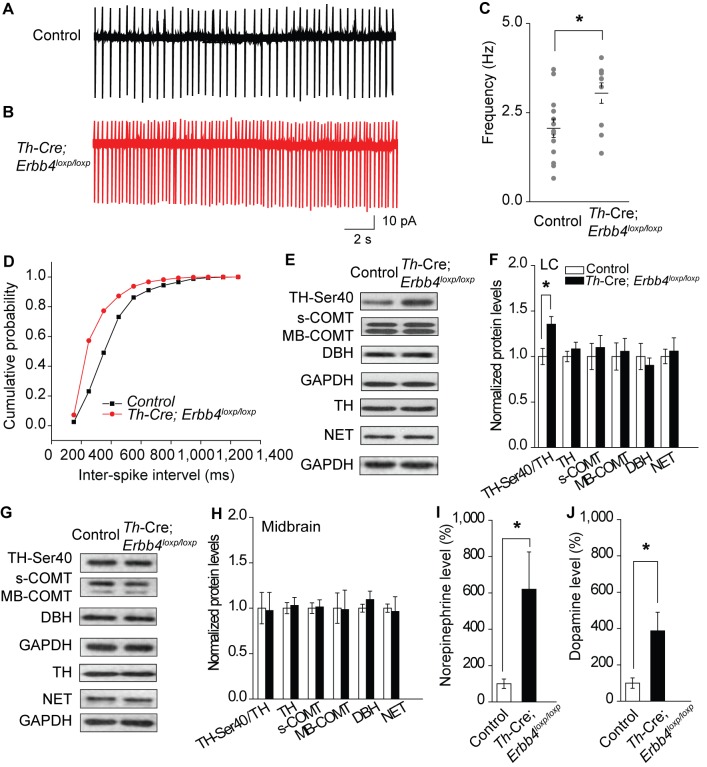


The corrected and original Figure 2-Source data 1 files are included in this correction notice as Source data 1 and Source data 2. 

The following contents have been amended accordingly:

In the “Results” session, the description of Figure 2A-D now reads:

“Consistent with previous studies, LC-NE neurons recorded from slices of the control mice exhibited a firing rate of 1.94 ± 0.18 Hz (Chandler et al., 2014; Jedema and Grace, 2004) (Figure 2A, C). However, the spontaneous firing rate of LC-NE neurons in the *Th-Cre;Erbb4^loxp/loxp^* mice was significantly increased (2.87 ± 0.18 Hz) (Figure 2B, C), and the inter-spike interval was significantly decreased (Figure 2D).”

The original version for reference was:

“Consistent with previous studies, LC-NE neurons recorded from slices of the control mice exhibited a firing rate of 2 ± 0.26 Hz (Chandler et al., 2014; Jedema and Grace, 2004) (Figure 2A, C). However, the spontaneous firing rate of LC-NE neurons in the *Th-Cre;Erbb4^loxp/loxp^* mice was significantly increased (3.04 ± 0.29 Hz) (Figure 2B,C), and the inter-spike interval was significantly decreased (Figure 2D).”

The legend of Figure 2C-D now reads:

“(C) Spontaneous firing frequency of LC-NE neurons increased in *Th-Cre;Erbb4^loxp/loxp^* mice. n = 10 from three mice (control); n = 8 from three mice (*Th-Cre;Erbb4^loxp/loxp^*). (D) Interspike intervals were calculated over 2 min of firing from each neuron. Interspike intervals were decreased in *Th-Cre;Erbb4^loxp/loxp^* mice compared with control mice. n = 10 from three mice (control); n = 8 from three mice (*Th-Cre;Erbb4^loxp/loxp^*).”

The original version for reference was:

“(C) Spontaneous firing frequency of LC-NE neurons was increased in *Th-Cre;Erbb4^loxp/loxp^* mice. n = 11 from three mice (control); n = 13 from three mice (*Th-Cre;Erbb4^loxp/loxp^*). (D) Interspike intervals were calculated over 2 min of firing from each neuron. Interspike intervals were decreased in *Th-Cre;Erbb4^loxp/loxp^* mice compared with control mice. n = 11 from three mice (control); n = 13 from three mice (*Th-Cre;Erbb4^loxp/loxp^*).”

In addition, we have provided more details of the experimental conditions for membrane property recordings for clarity and the relevant description under “Electrophysiology” subtitle in the “Materials and Methods” session now reads:

“For intrinsic membrane properties and spontaneous firing recordings, microelectrodes (3–5 MΩ) were filled with a solution containing 130 mM potassium gluconate, 20 mM KCl, 10 mM HEPES buffer, 2 mM MgCl·6 H_2_O, 4 mM Mg-ATP, 0.3 mM Na-GTP, and 10 mM EGTA; the pH was adjusted to 7.25 with 10 M KOH. 3 mins for stabilization. AP-V (50 μM, Tocris Bioscience), DNQX (30 μM, Tocris Bioscience) and picrotoxin (50 mM) were present in the bath solution for intrinsic membrane properties recordings. Spontaneous firing was recorded for at least 4 min for each neuron.”

The original version for reference was:

“For intrinsic membrane properties and spontaneous firing recordings, microelectrodes (3–5 MΩ) were filled with a solution containing 130 mM potassium gluconate, 20 mM KCl, 10 mM HEPES buffer, 2 mM MgCl·6 H_2_O, 4 mM Mg-ATP, 0.3 mM Na-GTP, and 10 mM EGTA; the pH was adjusted to 7.25 with 10 M KOH. 3 mins for stabilization. Spontaneous firing was recorded for at least 4 min for each neuron.”

Meanwhile, we replaced the representative trace of control (*Erbb4^loxp/loxp^*+AAV-GFP) in Figure 6A with a more typical one in the corrected version.

The corrected Figure 6 is shown below:

**Figure:**
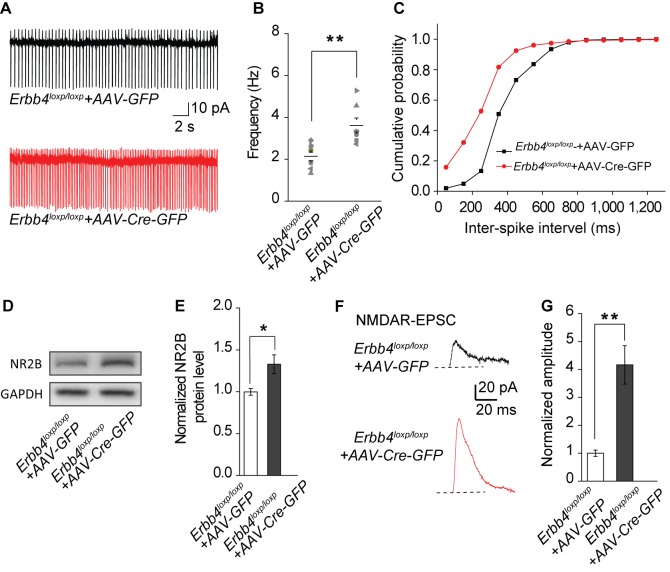


The original published Figure 6 for reference was:

**Figure:**
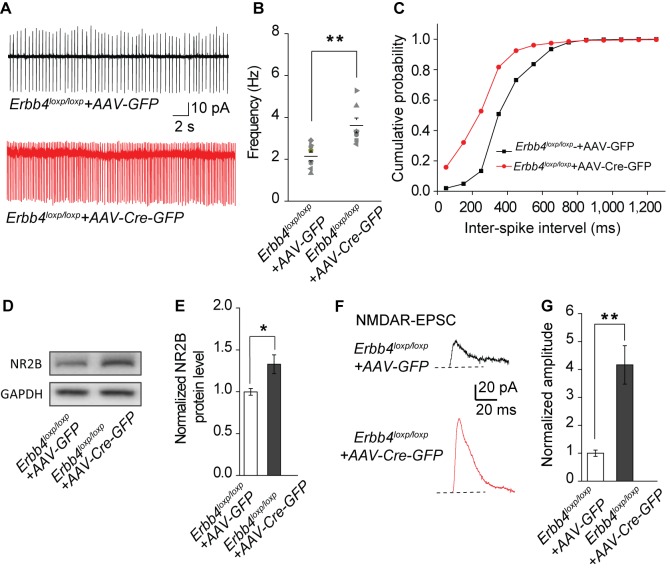


These corrections do not affect the results or conclusions of the original paper.

The article has been corrected accordingly.

## Additional files

Source data 1. Corrected version of Figure 2 - source data 1.

Source data 2. Original version of Figure 2 - source data 1.

